# Formulation of Polymeric Nanoparticles Loading Baricitinib as a Topical Approach in Ocular Application

**DOI:** 10.3390/pharmaceutics16081092

**Published:** 2024-08-20

**Authors:** Negar Beirampour, Paola Bustos-Salgado, Núria Garrós, Roya Mohammadi-Meyabadi, Òscar Domènech, Joaquim Suñer-Carbó, María José Rodríguez-Lagunas, Garyfallia Kapravelou, María Jesús Montes, Ana Calpena, Mireia Mallandrich

**Affiliations:** 1 Departament de Farmàcia i Tecnologia Farmacèutica, i Fisicoquímica, Facultat de Farmàcia i Ciències de l’Alimentació, Universitat de Barcelona, Av. Joan XXIII 29-31, 08028 Barcelona, Spain; nbeirabe7@alumnes.ub.edu (N.B.); paola2006@hotmail.com (P.B.-S.); rmohammo31@alumnes.ub.edu (R.M.-M.); jsuner@ub.edu (J.S.-C.); anacalpena@ub.edu (A.C.); 2 Institut de Nanociència i Nanotecnologia, Universitat de Barcelona (UB), 08028 Barcelona, Spain; 3 Department of Biochemistry and Physiology, Faculty of Pharmacy and Food Sciences, University of Barcelona, Av. Joan XXIII, 08028 Barcelona, Spain; mjrodriguez@ub.edu; 4 Department of Physiology, Institute of Nutrition and Food Technology (INyTA), Biomedical Research Center (CIBM), Universidad de Granada, 18100 Granada, Spain; kapravelou@ugr.es; 5 Department de Biologia, Sanitat i Medi Ambient, Facultat de Farmàcia i Ciències de l’Alimentació, Universitat de Barcelona, Av. Joan XXIII 29-31, 08028 Barcelona, Spain; mjmontes@ub.edu

**Keywords:** baricitinib, poly(lactic-co-glycolic acid) nanoparticles, poly(ε-caprolactone) nanoparticles, transcorneal permeation, ocular tolerance, ocular delivery

## Abstract

Topical ocular drug delivery faces several challenges due to the eye’s unique anatomy and physiology. Physiological barriers, tear turnover, and blinking hinder the penetration of drugs through the ocular mucosa. In this context, nanoparticles offer several advantages over traditional eye drops. Notably, they can improve drug solubility and bioavailability, allow for controlled and sustained drug release, and can be designed to specifically target ocular tissues, thus minimizing systemic exposure. This study successfully designed and optimized PLGA and PCL nanoparticles for delivering baricitinib (BTB) to the eye using a factorial design, specifically a three-factor at five-levels central rotatable composite 2^3+^ star design. The nanoparticles were small in size so that they would not cause discomfort when applied to the eye. They exhibited low polydispersity, had a negative surface charge, and showed high entrapment efficiency in most of the optimized formulations. The Challenge Test assessed the microbiological safety of the nanoparticle formulations. An ex vivo permeation study through porcine cornea demonstrated that the nanoparticles enhanced the permeability coefficient of the drug more than 15-fold compared to a plain solution, resulting in drug retention in the tissue and providing a depot effect. Finally, the in vitro ocular tolerance studies showed no signs of irritancy, which was further confirmed by HET-CAM testing.

## 1. Introduction

The eye is an exceptional organ of complexity. It is vulnerable to different disorders and diseases. Among these is uveitis, an autoimmune condition where abnormal immune responses target various structures within the eye [1]. These diseases require a systematic and multidisciplinary management strategy aimed at controlling inflammation, preserving visual function, and preventing further damage to ocular structures. Corticosteroids, such as prednisolone or dexamethasone, are commonly used as the initial therapeutic intervention to rapidly suppress inflammation [2]. In addition to corticosteroids, other immunosuppressive agents may be prescribed, such as methotrexate, azathioprine, mycophenolate mofetil, and cyclosporine. These medications work by modulating the immune response and preventing the production of inflammatory cells or chemicals [3]. In extreme cases, surgical interventions such as corneal transplants or retinal surgeries may be necessary, though their success can be compromised by immunological allograft rejection [4,5]. Research continues to focus on preventing graft rejection and improving graft survival [6,7].

Baricitinib (BTB), a Janus kinase (JAK) inhibitor, selectively inhibits Janus kinases 1 and 2, reducing inflammation, cellular activation, and the proliferation of key immune cells, thereby mitigating disease symptoms. BTB, administered orally, has shown good outcomes in treating refractory rheumatoid arthritis and atopic dermatitis [8]. In 2020, Yutaka Kaneko et al. examined the use of topical BTB for treating uveitis and dry eye disease, showing that BTB eye drops effectively reduced ocular inflammation and improved symptoms [9].

However, the corneal epithelium and tear fluid act as barriers, limiting drug penetration into the eye. These results in low bioavailability for ophthalmic drugs in aqueous solutions, exacerbated by rapid drug elimination due to reflex blinking and tear drainage, making it difficult to achieve therapeutic drug levels [10]. This presents an opportunity for nanomedicine, which can directly deliver drugs or genes to maximize therapeutic effect and minimize side effects. Nanoparticles (NPs) have shown promise in ocular drug delivery for immunosuppressive drugs. For example, Tacrolimus-loaded NPs have shown promise in treating dry eye syndrome and graft-versus-host disease [11,12], and cyclosporine A encapsulated in NPs, such as poly (lactic-co-glycolic acid) (PLGA) or chitosan NPs, have been studied to improve efficacy and prolong release for treating dry eye disease and ocular surface inflammation [13]. Further, Sirolimus-loaded NPs have been explored for corneal graft rejection and proliferative vitreoretinopathy [14], and Dexamethasone-loaded NPs have been investigated for treating uveitis and macular edema [15]. These NP-based systems offer advantages like improved drug stability, sustained release, and optimized bioavailability, which can reduce dosing interval. Enhancing the local delivery of immunosuppressive drugs aims to increase therapeutic efficacy while minimizing systemic side effects [16,17]. Further research is needed to optimize formulations and evaluate the safety and efficacy of NPs in ophthalmic applications.

This study aims to develop advanced BTB NPs using PLGA and polycaprolactone (PCL) for ocular delivery to manage inflammatory or immunological ocular diseases. The BTB NP formulations were optimized using a factorial design. The formulations were characterized in terms of physicochemical properties and morphology. The capacity of BTB to penetrate the cornea was evaluated through an ex vivo permeation study. The tolerability of the NPs was assessed via the HET-CAM test, corneal transparency, and histological analysis. Additionally, a microbiological study on the optimized formulations during their storage and possible use was conducted.

## 2. Materials and Methods

### 2.1. Materials

The BTB bulk powder ingredient was supplied by Henrikang Biotech Co., Ltd. (Xi’an, China). PLGA [poly (lactic-co-glycolic acid)] (Resomer^®^ RG 503H) was acquired from Boehringer Ingelheim (Ingelheim, Germany) and Poloxamer 188 (Lutrol^®^ F68) was acquired from BASF (Ludwigshafen, Germany). PCL [poly (ε-caprolactone)], with an average molecular weight of ~14,000 Da, and Acetone were acquired from Sigma–Aldrich (Barcelona, Spain). Transcutol^®^ was gifted by Gattefossé (Barcelona, Spain). The purified water used in all experiments was obtained from the Milli-Q^®^ Plus System (Millipore Corporation, Burlington, MA, USA) lab supply.

### 2.2. Biological Materials

The study utilized corneas sourced from the remains of pigs (a crossbreed of Landrace and Large White, weighing between 20 and 25 kg) that had been previously used in surgical procedures at a university. The Ethics Committee of Animal Experimentation at the University of Barcelona approved the use of these corneas (code 10619, approved on 10 January 2019). Immediately after the animals were slaughtered, the eyes were removed for corneal tissue dissection and then stored in a solution of artificial aqueous humor for transport to the laboratory. The corneas were then debrided and prepared for permeation experiments.

### 2.3. Factorial Design

To develop the NPs, a factorial design was conducted considering three-factor at five-levels-central rotatable composite design 2^3+^ star (Table 1 and Table 2). As dependent variables, physicochemical properties, such as average particle size (Z-ave) and polydispersity index (PDI), were chosen according to the central composite design matrix generated by Statgraphics^®^ Plus 5.1 software (Statpoint Technologies, Inc., Warrenton, VA, USA).

A total of 19 experiments for estimation of the pure error sum of squares were performed (Table 2). The observed experimental responses resulted from the individual influence and interactions of the three independent variables. To identify the significance of the effects and the interactions between them, analysis of variance (ANOVA) was performed for each parameter and *p* values pf less than 0.05 were considered to be statistically significant.

### 2.4. Preparation of the Selected Nanoparticles Loading BTB

To prepare the optimized NPs (B-PLGA-95, B-PLGA-75 and B-PCL NPs), the solvent displacement technique was used. A series of organic solutions was prepared by dissolving one the polymers (50 mg of PCL, 95 mg of PLGA, or 75 mg of PLGA) in 10 mL of acetone with 10 mg of BTB. Simultaneously, 200 mg of surfactant (P188) was dissolved in water adjusted to pH 5.5. The volumes of water were determined according to the formulations presented in Table 3. The previously prepared organic solution was added dropwise to the aqueous solution under moderate magnetic stirring at room temperature (25 °C). Then, the organic solvent evaporated overnight at room temperature (around 22 ± 2 °C) under ambient pressure (around 1 atmosphere). These conditions are based on standard laboratory procedures for evaporation techniques to remove organic solvents from nanoparticle suspensions.

### 2.5. Physicochemical Characterization of Nanoparticles

The physicochemical properties of the NPs were evaluated by measuring the pH, vesicle size (Z-ave), polydispersity index (PDI), and zeta potential (ZP). The Z-ave and PDI properties were determined at 25 °C using a Nano ZS Zetasizer^®^ (Malvern Instruments, Malvern, UK) via dynamic light scattering. ZP measures the surface charge of cells by evaluating the electrokinetic potential; this characterizes the electrical potential of the double layer on the cell surface and serves as an indirect measure of physical stability. The pH was measured at room temperature using a Micro pH 2001 pH meter (Crison Instruments SA, Alella, Spain) [18]. All measurements were conducted in triplicate and are reported as mean ± standard deviation.

#### 2.5.1. Encapsulation Efficiency (EE)

The encapsulation efficiency (EE%) was determined using an indirect method. Initially, 0.5 mL of nanoparticle suspension was placed into a centrifugal filter unit (Amicon Ultra, Millipore, Billerica, MA, USA) and centrifuged at 11,000 r.p.m. (12,000× *g*) at 4 °C for 15 min. The filtered solution, containing non-encapsulated BTB, was then analyzed using high-performance liquid chromatography (HPLC) [19]. The HPLC system utilized a Chromatograph Waters Alliance 2695 with a Fluorescence Jasco FP-1520 detector (Waters, Milford, CT, USA), employing an excitation wavelength of 310 nm and an emission wavelength of 390 nm. A Symmetry C18 column (3.5 µm, 4.6 × 75 mm) was used with a mobile phase consisting of Ammonium Formate (10 mM, pH 7) and ACN (75:25 *v*/*v*) delivered at a flow rate of 1 mL/min under isocratic elution conditions. With an injection volume of 10 µL, the quantification of BTB was validated within a range of 0.06–1 µL/min. Finally, the encapsulation efficacy (%) was calculated by dividing the amount of non-encapsulated BTB from the total amount of BTB in the NPs and multiplying by 100, as described in (Equation (1)):

EE% = (A_non-e_/A_total_) × 100 (1)

A_non-e_ is the amount of BTB non-encapsulated and A_total_ is the total amount of BTB in the NPs.

#### 2.5.2. Morphological Study of Nanoparticles

The transmission electron microscopy (TEM) was used for the morphological and structural examination of NPs. First, one drop of each NP solution was adsorbed into carbon-coated copper grids for 1 min. Then, they were stained with UranyLess EM Stain (EMS) for 1 min. The excess liquid was manually blotted from the edge of the grids. The sample was observed in a JEOL 1010 (JEOL Inc., Peabody, MA, USA) microscope equipped with a tungsten filament. Finally, images were acquired at 120 kV at room temperature with a 1376 × 1024 pixels CCD Mega view camera [20].

#### 2.5.3. Extensibility of Nanoparticles

To assess the spreading behavior of the NPs, a 50 µL volume of each formulation was added to a steel plate circle with a diameter of 10 cm, with a glass plate weighing 26 g being placed on top. Increasing standard weight pieces (0, 10, 20, 50, 100 g) were added on top of the upper glass plate for 1 min each. The diameter of the spread area was recorded for each formulation and tested in triplicate at room temperature. The resulting increase in surface area (mm^2^) of the NPs was plotted as a function of the increasing weights applied [21]. The two-site binding parabola model described in Equation (2) was best fitted to the formulations measured.

Y = A + B × X + C × X^2^, (2)

A, B and C are coefficients, X the weight in g, and Y the extensibility in cm^2^.

#### 2.5.4. Rheological Characterization

Rheological measurements were performed with a Haake Rheostress^®^ 1 rheometer (Thermo Fisher Scientific, Karlsruhe, Germany) connected to a thermostatic circulator Thermo^®^ Haake Phoenix II + Haake C25P. Steady-state measurements were addressed with a plate-and-cone geometry (C60/2°Ti: 60 mm diameter, 2° angle). The shear stress (τ) was measured as a function of the shear rate (γ). Viscosity curves (η = f(γ)) and flow curves (τ = f(γ)) were recorded at 25 ± 0.1 °C. The shear rate ramp program included a 3 min ramp-up period from 0 to 100 s^−1^, a 1 min constant shear rate period at 100 s^−1^, and a 3 min ramp-down from 100 to 0 s^−1^. Representative mathematical models were fit to flow curves as the best descriptive model. Selection of the best fitting was based on the correlation coefficient (observed vs. predicted) and chi-square value. Steady-state viscosity (η, m·Pa s) was determined from the constant shear section at 100 s^−1^.

#### 2.5.5. Stability of Nanoparticles

The physicochemical stability of NPs was assessed at 25 °C by dynamic light scattering analysis (DLS) with Z-sizer (Malvern Instruments, Malvern, UK) profiles. The optimized NPs were divided into two batches and stored at different temperature conditions according to the established guidelines at 5 ± 3 °C and 25 ± 2 °C, with a relative humidity of 60% ± 5% [22]. The values of Z-Ave and PDI were recorded at 0 d, 7 d and 30 d after the NP preparation.

### 2.6. Antimicrobial Challenge Test

During the development phase of a pharmaceutical formulation for ophthalmic use, it must be demonstrated that the formulation provides adequate protection against adverse effects that may occur from microbial contamination or growth during storage and use [19,23]. The Challenge Test involved the deliberate contamination of the formulations, in their final containers, with a determined inoculum of suitable microorganisms (Table 4). All the microbial strains (*Pseudomonas aeruginosa*, *Staphylococcus aureus*, *Candida albicans* and *Aspergillus brasiliensis*) used in this study were acquired from the collection at the microbiology laboratory of the Faculty of Pharmacy and Food Sciences, University of Barcelona. Tryptone Soya Agar (TSA; Ref: CM0131B, Oxoid, UK) was inoculated with bacteria and incubated for 18–24 h at 37 ± 2 °C. *C. albicans* and *A*. *brasiliensis* were grown on Sabouraud Dextrose Agar (SDA; Ref: CM0041B, Oxoid, UK) at 25 °C for 48 h and 5 d, respectively. For the collection of subcultures and preparation of bacterial and fungus suspensions that will be used to inoculate the formulations, a sterile saline test solution (NaCl 0.9% *w*/*v*) was applied to reach the final concentration of 10^8^ CFU/mL (CFU = colony forming units). In the case of the A. *brasiliensis* culture, a sterile saline test solution containing 0.05% (*v*/*v*) of polysorbate 80 was used. 

Each NP formulation was transferred to containers in triplicate and individually inoculated with 1% of each microbial test suspension separately in order to make the final concentrations in the samples match those indicated in the pharmacopoeia (10^5^–10^6^ CFU/mL) [24]. The NP formulations were kept at room temperature (20–25 °C) and protected from light during the withdrawal of samples from the container (1 mL). At specified time intervals, samples were taken and microorganisms were counted and recorded (Table 5). The samples were neutralized using Beerens (Ref. 02-257 Scharlab, Barcelona, Spain). The average log10 CFU/mL were used to quantify the results. The formulations were found to be adequate if, under the test conditions, there was a significant decrease or, depending on the case, there was no increase in the number of microorganisms after the established times and temperatures (Table 5).

Criteria A expresses the recommended effectiveness. In justified cases where it is not possible to meet the A criteria (for example, due to an increased risk of adverse reactions), then B criteria should be met.

### 2.7. Ex Vivo Cornea Permeation Assays

**Figure 1:**
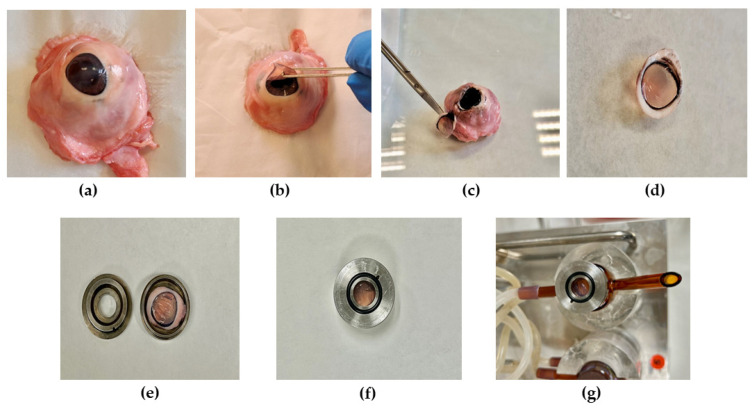
Isolation of the cornea and assembly on the Franz cell with 0.64 cm^2^ diffusional area for the ex vivo transcorneal permeation study: (**a**) excised porcine eyeball; (**b**,**c**) isolation of the cornea from the eyeball; (**d**) isolated porcine cornea; (**e**,**f**) fasten of the cornea in the donor compartment; and (**g**) assembly of the donor compartment on the Franz cell.

Ex vivo studies were performed using porcine corneas (see Section 2.2 biological material). The experiments were conducted in independent vertical Franz diffusion cells with a diffusional surface area of 0.64 cm^2^. We applied 200 µL of NPs, either B-PLGA-95, B-PLGA-75, and B-PCL, in the donor chamber (in sextuplicate per each formulation). As a control, a solution of BTB in phosphate buffer pH 7.4 was included in the study. Cornea tissues were positioned between the two compartments of a Franz cell with the corneal side facing upwards, towards the donor chamber, with the other side staying in contact with the receptor medium. The donor chamber was covered with a parafilm to prevent evaporation during the study. Phosphate buffered saline (PBS), namely Transcutol^®^ (1:1), was used as the receptor medium. This was necessary due to the low solubility of BTB on Transcutol^®^ [25]. The permeation study was conducted for 6 h at 32 ± 0.5 °C under continuous stirring to maintain sink conditions. At each sampling interval of up to 6 h, a volume 200 μL of receptor medium was withdrawn and an equal volume of fresh Transcutol^®^ was added. Samples were analyzed by HPLC (see Section 2.5.1) with a fluorescence detector to determine the cumulative amount of the drug that was permeated. Figure 1 shows the isolation of the cornea from the eyeball and the preparation of the Franz cells.

Following the ex vivo permeation study, the drug retained in the corneas was extracted as follows: after disassembling the corneas from the diffusion cells, the NPs on the corneal surface were eliminated by rinsing them with distilled water. The permeation area was isolated, weighed, pricked with a thin needle, submerged in 1 mL of Transcutol^®^, and sonicated in an ultrasonic water bath for 15 min. The resultant solution was filtered and quantified by HPLC (see Section 2.5.1) to obtain the amount retained in the tissues (*Q_ret_*) according to Equation (3):

(3) $$Q_{ret}=\frac{Q_{ext}}{A\times W\times R}$$

*Q_ext_* is the amount of drug retained in the cornea (μg), *A* is the Franz cell diffusion area (cm^2^), *W* is the weight of the cornea (mg), and *R* is the recovery of BTB in each tissue [26,27].

Permeation parameters such as the permeability coefficient (*K_p_*, cm/h), flux (*J*, μg h^−1^cm^−2^), partition and diffusion parameters (*P*_1_ and *P*_2_), and the lag time (*T_l_*, h) were calculated from the ex vivo permeation study. The permeation profiles were structured by plotting the cumulative amount of (μg) permeated per surface area of the corneal tissue (cm^2^) versus time (h). In the plot, the *T_l_* is the intercept with the *X*-axis (time) and the flux is the slope of the linear part of the permeation profile calculated by linear regression analysis (GraphPad Prism 5 software, Inc., San Diego, CA, USA). The permeability coefficient was calculated through Equation (4), as follows:

(4) $$K_{p}=\frac{Jss}{C_{0}}$$

*Jss* (μg/h/cm^2^) is the flux across the cornea and *C*_0_ (μg/cm^3^) is the initial amount of formulation tested in the donor compartment. It is assumed that, under sink conditions, the drug concentration in the receiver compartment is negligible compared to that in the donor compartment [28]. Other permeation parameters were calculated according to the following equations:

(5) $$P_{2}=\frac{1}{6.T_{l}}$$

where *P*_2_ is the diffusion coefficient and *T_l_* is the lag-time.

(6) $$P_{1}=\frac{K_{p}}{P_{2}}$$

where *P*_1_ is the partition coefficient, *P*_2_ is the diffusion coefficient, and *K_p_* is the permeability coefficient.

The ratio of the permeability coefficients between NPs and the BTB solution led to calculating the permeation enhancement ratio (*ER*) with Equation (7) [29], as follows:

(7) $$ER=\frac{{Kp\;}_{nanoparticle}}{{Kp}_{\;solution}}$$

One method used to assess the integrity of the cornea is to determine the percentage levels of corneal hydration (HL), which was obtained through Equation (8), as follows:

HL (%) = (1 − *W_b_*/*W_a_*) × 100 (8)

Upon completion of the permeation study, the corneas were recovered from the Franz cells and weighed; this weight determined the wet corneal weight (*W_a_*). The corneas were then desiccated until a constant weight was reached. After desiccation at 80–100 °C for 6 h, samples were weighed again to determine the dry corneal weight (*W_b_*) [30]. 

### 2.8. In Vitro Tolerance Study

#### 2.8.1. Hen’s Egg Test on the Chorioallantoic Membrane (HET-CAM)

The ocular potential irritancy of NPs was determined by observing the adverse changes that occurred in the chorioallantoic membrane (CAM) of fertilized eggs after exposure to the tested NPs. Eggs were incubated for 9 d, after which non-viable or defective eggs were discarded. The shell on the air cell portion was removed and the inner membrane was extracted after being washed and hydrated with PBS to reveal the CAM. Then, 300 μL of the test NPs were applied to the CAM and reactions were observed within 300 s. One egg was treated with 0.1 N sodium hydroxide as the positive control and another with 0.9% NaCl solution as the negative control. The endpoints to be observed were bleeding (bleeding from vessels), vascular lysis (breakdown of blood vessels), and coagulation (intravascular and extravascular protein denaturation) [30,31]. The severity of vascular damage observed in the chorioallantoic membrane is an indication of the product’s potential to damage mucosal membranes in vivo, assuming that there is a correlation between the severity of damage and the speed at which it occurs [26]. The time of the appearance and the intensity of any reactions that occur within 5 min were recorded according to the INVITTOX protocol [26]. Then, the eye irritation score (*IS*) was calculated using Equation (9) and the degree of potential irritation for each formulation was established based on the criteria defined by the INVITTOX protocol (Table 6).

(9) $$IS=\frac{301-H}{300}\cdot 5+\frac{301-L}{300}\cdot 7+\frac{301-C}{300}\cdot 9$$

*H* is the time (s) to the beginning of hemorrhage, *L* is the time (s) to lysis, and *C* is the time (s) to coagulation.

#### 2.8.2. Cornea Transparency

The corneas were carefully isolated from the eyeball with a scalpel and rinsed with PBS pH 7.4. One eye from the same donor served as a negative control while the other eye was exposed to either ethanol (positive control) or one of the NPs. Three animals were included per group, which were the following: (i) ethanol; (ii) B-PLGA-95; (iii) B-PLGA-75 and (iv) B-PCL. The corneas were mounted on the Franz cells, and 2 drops of the following solutions were applied to the cornea: physiological saline solution as negative control, ethanol as positive control, and the colloidal suspensions of B-PLGA-95, B-PLGA-75 and B-PCL NPs. The exposure time was 2 h, and 1–2 drops of physiological saline solution was applied to the cornea every 5–10 min to simulate the tear clearance. After two hours, the corneas were demounted from the Franz cells and evaluated for transparency by measuring the transmittance with a Nanodrop^®^ spectrophotometer TM 2000 (Thermo^®^ Fisher Scientific, Waltham, MA, USA). Samples were cleaned with saline solution to remove any contaminants and carefully dried under a stream of N_2_. The transmittance of each sample was measured in the range of 380–780 nm in at least 3 different regions of the cornea near its center to evaluate any changes in corneal transparency caused by the NPs.

#### 2.8.3. Cornea Histology Study

To determine if the formulations had an impact on the structure of the cornea, the same procedure used for the cornea transparency evaluation was followed. For the histological examination, the three different NPs formulations (B-PLGA-95, B-PLGA-75 and B-PCL) were applied to the corneal tissues while distilled water served as the negative control. The tissues were exposed to the formulations for 3 h, followed by processing for hematoxylin and eosin staining [31]. To prepare the tissues for histology, the corneas were immersed in 4% buffered paraformaldehyde solution for 24 h to fix the tissues, then dehydrated with increasing concentrations of ethanol. Finally, the corneas were embedded in paraffin, cut into sections of 5 μm, and stained with hematoxylin and eosin; DPX was used as the mounting medium (Sigma–Aldrich). The histological samples were analyzed under a microscope Olympus BX41 and camera Olympus XC50; (Olympus, Hamburg, Germany) in a blinded fashion to evaluate any changes in corneal structure caused by the nanoparticle formulations.

## 3. Results

### 3.1. Factorial Design

A factorial design was conducted to optimize the development of NPs with two polymers, PLGA and PCL. The evaluation of the effects considered three factors (amount of polymer, amount of surfactant, and volume of the aqueous phase) and used the responses (Z-ave and PDI) to select the optimized NP composition from a total of 19 formulations with each polymer. Table 7 shows the mean particle size and polydispersity index for PLGA-based NPs and Table 8 reports the results obtained for the responses for the PCL-based NPs.

**Figure 2:**
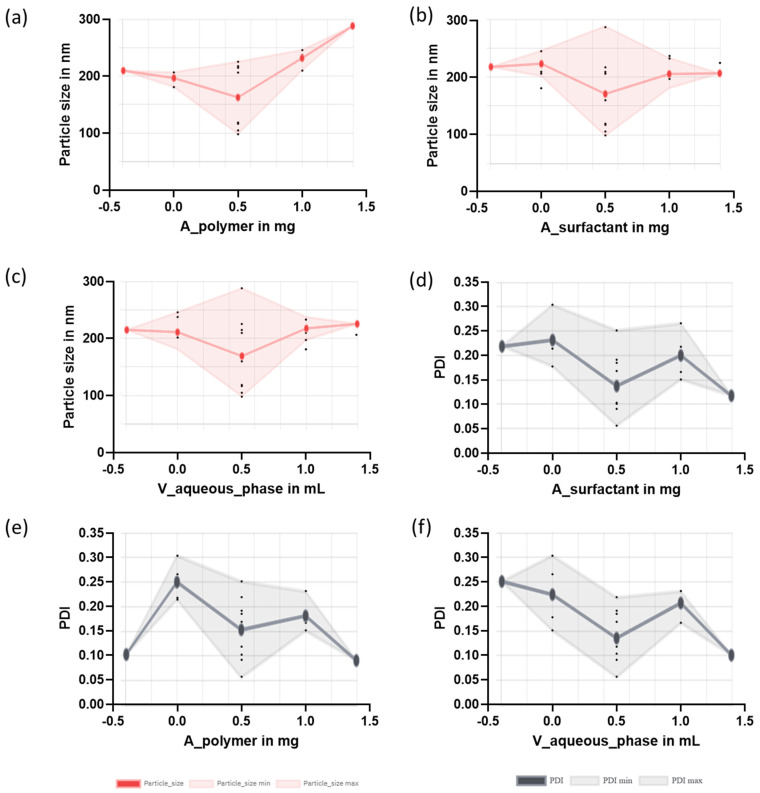
Main effects of the factors on the responses for the PLGA-based NPs. Effects on the particle size (red panels): (**a**) amount of polymer on the particle size; (**b**) volume of the aqueous phase on the particle size; (**c**) amount of surfactant on the particle size. Effects on the PDI (grey panels): (**d**) amount of surfactant on the PDI; (**e**) amount of polymer on the PDI; (**f**) volume of the aqueous phase on the PDI. The *x*-axis is given as levels which are low (0), centered (0.5), high (1), and axial (−0.39; 1.39).

The mean particle size for the PLGA-based NPs was within the range of 98.20 and 288.20 nm. The polydispersity index (PDI) indicates the degree of uniformity in the size distribution of the NPs. For polymeric NPs, values of PDI of up to 0.2 are considered acceptable. Most of the NPs exhibit PDI values below 0.2, indicating that the obtained NPs were monodisperse colloidal systems [32]. In general, a low amount of surfactant led to higher PDI values. Figure 2 illustrates the effects of several factors (amount of polymer, surfactant, and volume of the aqueous phase) on particle size and PDI for the PLGA-based NPs.

In ocular delivery, the size and distribution of the particles are critical features. Since large particles should irritate and can cause discomfort to the eye [32,33], the optimization of the NPs was focused on obtaining small NPs with narrow distribution sizes [34]. F9 was selected among the 19 formulations prepared, which corresponded to the centered level for the three factors. In addition, a modified F5 formulation was also prepared (with an increased amount of surfactant) taking into account that the central point of surfactant led to small NPs and narrow PDI. The values in Table 2 informed the composition of the modified F5 formulation, which was further characterized and evaluated in subsequent studies.

**Figure 3:**
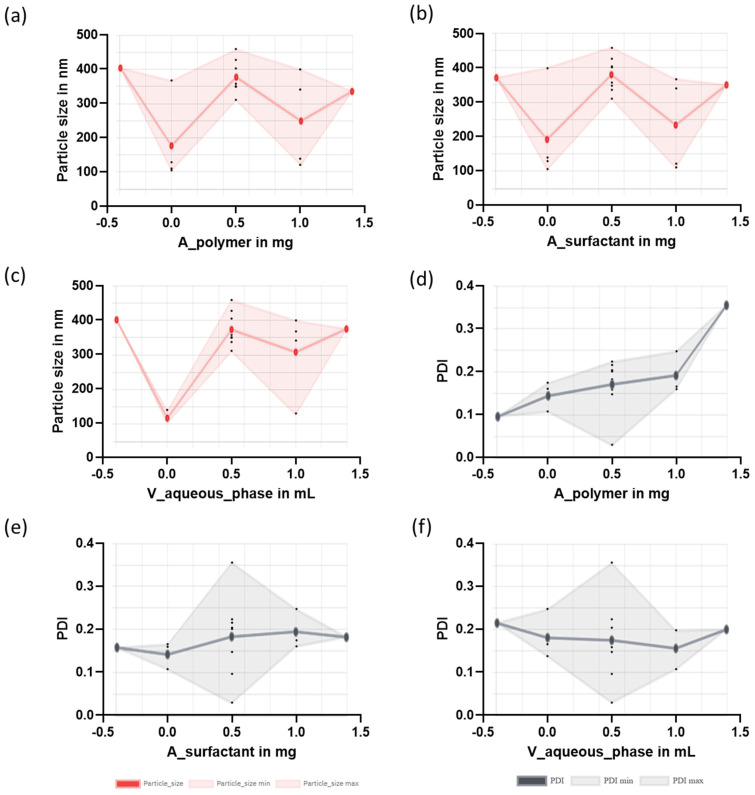
Main effects of the factors on the responses for the PCL-based NPs. Effects on the particle size (red panels): (**a**) amount of polymer on the particle size; (**b**) amount of surfactant on the particle size; (**c**) volume of the aqueous phase on the particle size. Effects on the PDI (gray panels): (**d**) amount of polymer on the PDI; (**e**) amount of surfactant on the PDI; (**f**) volume of the aqueous phase on the PDI. The *x*-axis is given as levels: low (0), centered (0.5), high (1) and axial (−0.39; 1.39).

Figure 3 shows the effects of the factors (amount of polymer, surfactant and aqueous phase) on the responses (particle size and PDI) for the PCL-based NPs. A low amount of polymer and surfactant and a small volume of the aqueous phase led to the smallest particle size. Low PDI values were found for the formulations composed of low amounts of polymer. Based on the results, the selected formulation for PCL-based NPs was F5 (Table 8), which corresponded to the low levels for the three factors (polymer, surfactant, and aqueous phase).

**Figure 4:**
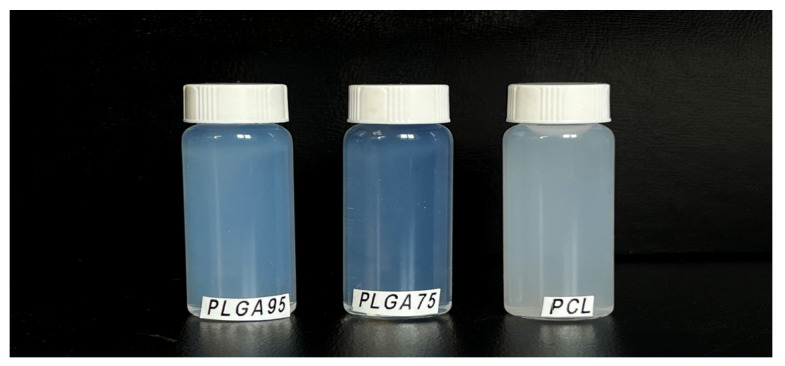
Physical appearance of the colloidal suspension of NPs.

Thus, further characterization and investigations were conducted on the selected formulations F9-PLGA, modified F5-PLGA and F5-PCL, which corresponded to the formulations indicated in Table 3 (B-PLGA-95, B-PLGA-75 and B-PCL, respectively). Figure 4 shows the suspension of selected NPs.

### 3.2. Nanoparticle Physicochemical Characterization

Particle size is a crucial parameter for ocular drug delivery systems, as it can affect the risk of irritation and discomfort [35]. The particle size of all the formulations that were developed were suitable for ocular administration, featuring diameters smaller than 250 nm (Table 9). Additionally, the polydispersity index (PDI) values for all NPs were less than 0.1, indicating a narrow size distribution [36]. Table 9 presents the physicochemical characteristics of the B-PLGA-95, B-PLGA-75, and B-PCL NPs. Despite the relatively low zeta potential (ZP) values, between −20 and −28 mV, the nanoparticles were shown to be stable for at least one month since they exhibited minor variations in particle size and PDI over time (Section 3.2.5). Zeta potential is a key parameter that influences particle stability; higher absolute ZP values generally indicate better stability due to increased electrostatic repulsion [37]. However, even with ZP values of around −20 mV, the formulations remained stable due to other contributing factors. The narrow size distribution, indicated by low PDI values, and the small particle sizes help maintain stability by reducing the likelihood of particle aggregation [38]. Moreover, all formulations had a pH of approximately 6.8, which is biocompatible with the ocular environment and further supports the stability and safety of these formulations for ocular use [39,40].

#### 3.2.1. Encapsulation Efficiency

The entrapment efficiency provides an insight into the amount of BTB that is effectively loaded in the NPs. Typically, an excellent drug carrier should have high entrapment efficiency (EE). High EE (above 70%) can increase the efficacy of the drug delivery system and decrease the side effects of the drug [41]. The EE of the B-PLGA-95, B-PLGA-75, and B-PCL NPs were measured in the range of 65.63 ± 3.87, 27.39 ± 2.05, and 77.82 ± 5.33, respectively (Table 9). The highest drug entrapment (77.82 ± 5.33%) was found in the case of B-PCL NPs. The large amount of BTB formulated in this sample is supposed to prevent the drug’s diffusion from the polymeric core, thereby enhancing the entrapment of the drug [42].

#### 3.2.2. Morphological Study

**Figure 5:**
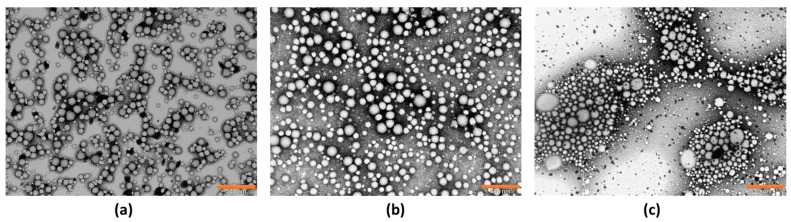
Transmission electron microscopy (TEM) images of NPs of (**a**) B-PLGA-95 NPs, (**b**) B-PLGA-75 NPs, and (**c**) B-PCL NPs. Bar length (**a**,**b**) 500 nm, (**c**) 1 µm.

The appearance, droplet, and particle size of nanoparticle formulations B-PLGA-95, B-PLGA-75, and B-PCL NPs were evaluated. The NPs were spherical in shape, and the TEM analysis confirmed the size distribution of NPs obtained by dynamic light scattering. As shown in Figure 5, droplets were nearly uniformly distributed in B-PLGA-95 and B-PLGA-75 formulations. However, B-PCL showed larger particles and a less uniform system compared to the other formulations. The results indicated that both the B-PLGA-95 and B-PLGA-75 NPs evaluated in this study were the most suitable for ophthalmic applications [43].

#### 3.2.3. Extensibility Studies

**Figure 6:**
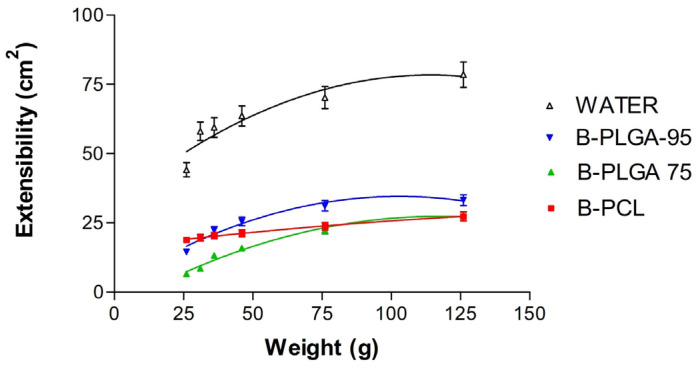
The scheme shows how all NPs follow the same mathematical fit models with their respective parameter values for extensibility (expressed in cm^2^ by weight (Mean ± SD) (*n* = 3)).

All of the formulations were in accordance with the two-site binding parabola model (Figure 6). Extensibility values increase with loading weight. The B-PLGA-95 NPs were the most extensible but did not exhibit significant statistical differences with B-PLGA-75 nor B-PCL NPs. Water was used as the reference.

Table 10 reports the predicted values of the parameters according to the two-site binding parabola model, which was the mathematical model that best described the extensibility behavior of the NPs.

#### 3.2.4. Rheological Characterization

**Figure 7:**
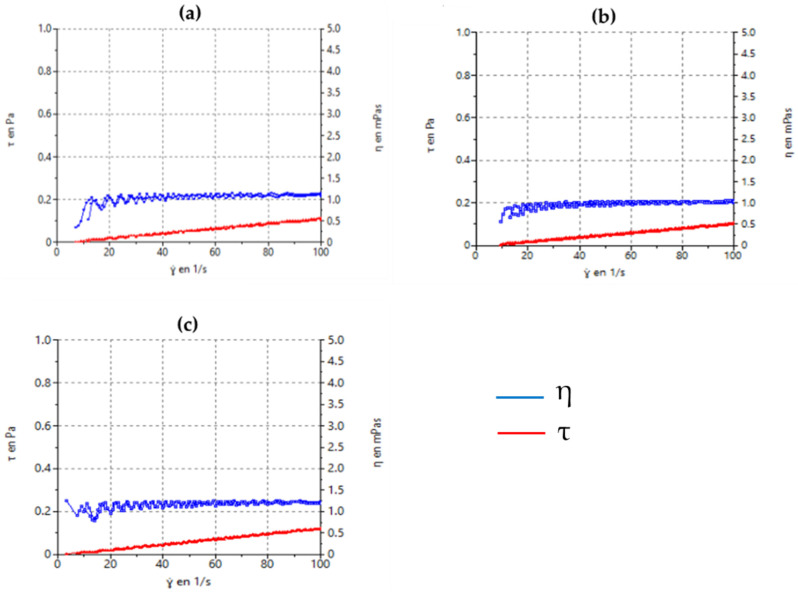
Rheograms of the tested formulations. (**a**) B-PLGA-95 NPs, (**b**) B-PLGA-75 NPs, (**c**) B-PCL NPs. The red line corresponds to the flow curve (τ) while the blue line represents the viscosity curve (η).

Steady-state rheological measurements as a function of shear rate are shown in Figure 7. All formulations exhibited typical Newtonian behavior in which the shear stress to shear rate relationship (flow curve) was linear, with an absence of thixotropy, and the viscosity remained constant. Viscosity values at 100 s^−1^ were 1.22 ± 0.02, 1.03 ± 0.01, and 1.09 ± 0.02 mPa·s for B-PCL, B-PLG-75, and B-PLGA-95 NPs, respectively.

#### 3.2.5. Stability of Nanoparticles

The physical stabilities of NPs were evaluated at two different temperatures upon formation (25 and 4 °C) for 30 d (last time point tested). NPs showed no physical changes, such as discoloration during the testing period. A slight increase in the NP size was observed for all the NPs stored at both temperatures, and although these differences were statistically different, they were minor changes that did not indicate particle aggregation. The same was observed for the PDI, i.e., a slight increase in the value over time with statistical differences, but no differences that were relevant to the stability since the NPs showed values of PDI below 0.2. Results are shown in Table 11.

### 3.3. Antimicrobial Challenge Test

Table 12 shows the results in CFU/mL of the antimicrobial Challenge Test for all the tested formulations. Based on the results, the B-PLGA-95 NP formulation meets criterion B. The B-PLGA-75 NPs met criterion A in terms of logarithmic reduction for both bacterial and fungal inoculum at the indicated times. In the case of the B-PCL formulation, the acceptance criteria were not met. The B-PCL NPs were compatible with *P. aeruginosa* but not with *S. aureus*, *C. albicans*, or *A. brasiliensis.* It would be necessary to add a suitable preservative to prevent growth or limit microbial contamination during storage and use [41]. 

### 3.4. Ex Vivo Permeation

The permeation study parameters of the studied NPs are depicted in Table 13. B-PLGA-95 NPs achieved the maximum flux, followed by B-PCL NPs, and B-PLGA-75 NPs. B-PLGA-95 NPs also exhibited the highest permeability *K_p_*. The *T*_1_ parameter indicates the time required for reaching the steady state; this time is inversely proportional to the drug’s diffusivity through the cornea. All NPs had a similar *T*_1_ between them. One of the factors, which controls the drug permeability between the formulation and the cornea, is the partition coefficient (*P*_1_). It is directly proportional to the distribution of the drug in the cornea. The greater the value of *P*_1_, the higher the affinity of the drug to the tissue. Thus, once again, B-PLGA-95 NPs were shown to have the highest distribution of NPs. In the case of the diffusion parameter *P*_2_, the results had a similar tendency.

The NPs composed of PLGA led to lower amounts of baricitinib retained in the cornea than the ones prepared with PCL, being about one-tenth B-PLGA-75 with respect to B-PLGA-95. Thus, when the NPs are exposed to the corneal surface, they form a drug reservoir from which BTB is directly delivered. The drug depot produced with BTB entrapped into NPs guarantees sustained permeation.

The hydration levels of the corneas were measured at the end of the ex vivo transcorneal permeation study to evaluate the integrity of the cornea. The values obtained were 77.1 ± 0.2%, 78.4 ± 0.5%, and 79.0 ± 0.3% for B-PLGA-95, B-PLGA-75, and B-PCL NPs, respectively.

### 3.5. In Vitro Tolerance Study

#### 3.5.1. Hen’s Egg Test on the Chorioallantoic Membrane (HET-CAM)

**Figure 8:**
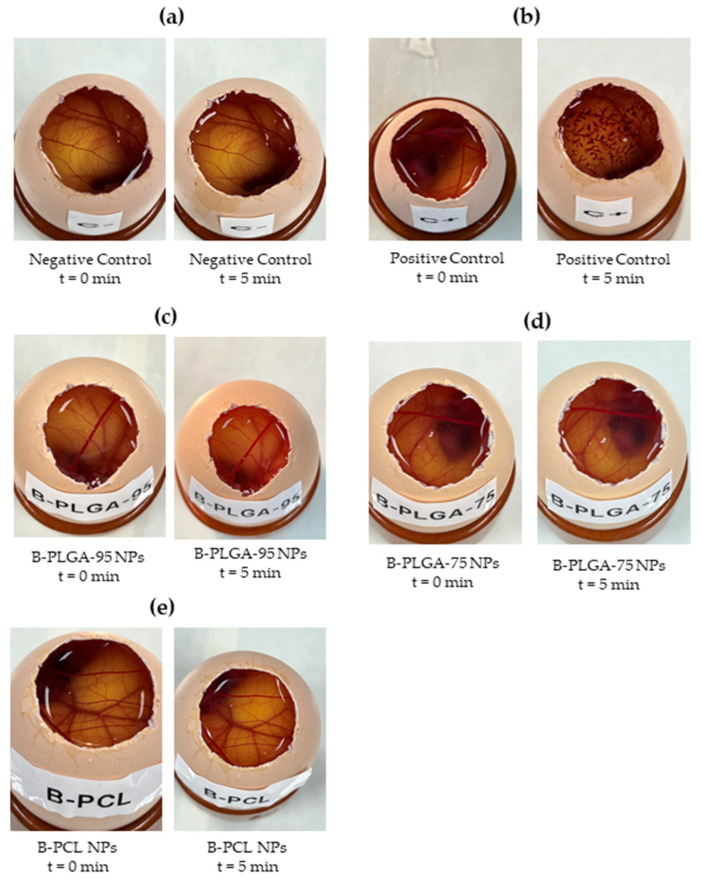
Evaluation of the irritant effect of the formulations by HET-CAM; (**a**) negative control (saline solution), (**b**) positive control (sodium hydroxide solution 0.1 N), (**c**) B-PCL nanoparticles, (**d**) B-PLGA-75 nanoparticles, and (**e**) B-PLGA-95 nanoparticles.

To determine if the nanoparticle formulations were well tolerated for ocular use, we conducted a HET-CAM in vitro test. The irritation score calculated for positive control was 20.48 ± 1.96. On the contrary, when the different NPs were tested, including B-PLGA-95, B-PLGA-75, and B-PCL formulations with 0.9% NaCl (negative control), no signs of ocular irritancy, such as coagulation, vascular lysis, or hemorrhage, were detected within 5 min. The irritancy score was below 0.1, demonstrating the safety of the NP formulations for ocular use since no reaction occurred during the test period (Figure 8).

#### 3.5.2. Cornea Transparency

**Figure 9:**
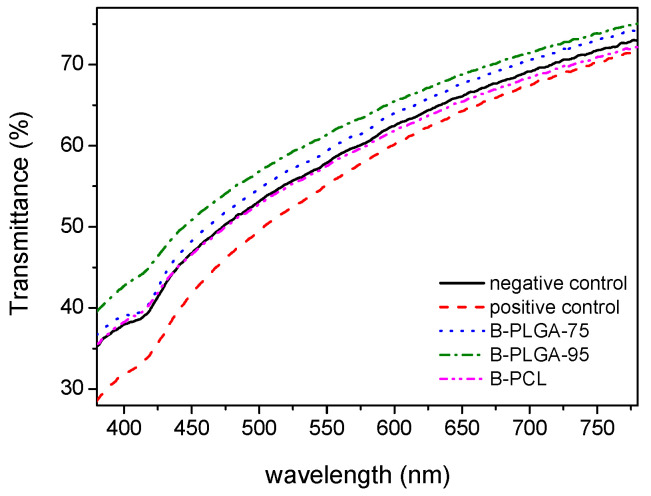
Transmittance values as a function of wavelength in the visible region for the different groups: Ethanol as the positive control, physiological saline solution as the negative control, and the treated corneas with B-PLGA-75, B-PLGA-95, and B-PCL NPs.

In Figure 9, transmittance values are shown as a function of wavelength for the different treated corneas. As expected, the positive control showed the lowest transmittance values in the range studied. On the one hand, corneas treated with B-PLC NPs or B-PLGA-75 NPs presented a clearness quite similar to the negative control, especially in the range of 380–480 nm. On the other hand, B-PLGA-95 NP-treated corneas showed higher transmittance values than the negative control, suggesting a possible improvement of corneal tissue clarity.

#### 3.5.3. Cornea Histological Study

**Figure 10:**
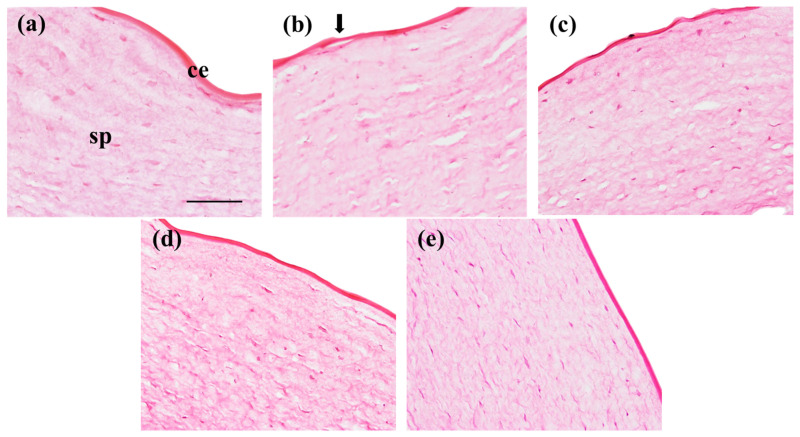
Cornea sections stained with hematoxylin and eosin. Cornea was treated with the following solutions: (**a**) serum (negative control conditions); (**b**) ethanol (positive control); (**c**) B-PLGA-95 NPs; (**d**) B-PLGA-75 NPs; and (**e**) B-PCL NPs. ce: corneal epithelium (non-keratinized stratified squamous epithelium); sp: substantia propria; arrow indicates disruption of ce. Magnification = 200×, scale bar = 100 µm.

The treatment of the cornea with ethanol induced a disruption of the corneal epithelium that is noticeable in Figure 10b. In negative control conditions, serum did not harm the cornea, as can be seen in Figure 10a. Similarly, the B-PLGA-75 and B-PCL formulations (Figure 10d,e) showed no damage to the corneal epithelium or the substantia propria. In the case of B-PLGA-95 (Figure 10c), the epithelium was not altered; however, the substantia propria showed some dilatations of the tissue that are comparable to the cornea treated with ethanol.

## 4. Discussion

Three variations of NPs containing BTB were developed for use as immunosuppressive therapy for ocular diseases. NPs have great potential as drug carriers for eye treatments with improved drug absorption in the eye compared to eye drop solutions due to the slower elimination rate of the particles [42]. NPs are an effective solution for ocular drug delivery because of their small size, which causes minimal irritation, and their ability to provide a sustained release, reducing the need for frequent drug administration [40]. The NPs developed with both quantities of PLGA have a size smaller than 85 nm and a polydispersity ≤ 0.1, with B-PLGA-75 being the smallest. While the B-PCL NPs have the larger size, they are still suitable for the ocular administration [43]. The ability of NPs to transport drugs is dependent on their size, with smaller particles having a higher permeability [44]. Since smaller particles are better tolerated by patients than larger ones, the PLGA NPs may offer a more comfortable and long-lasting eye delivery system. A high ZP value increases the repulsive forces of NPs, resulting in better stability [45]. B-PLGA-95 is the formulation with the highest ZP, followed by B-PLC, while the lowest formulation is B-PLGA-75. The pH is similar for all NPs, with a value close to the ideal pH for ophthalmic formulations (that of tear fluid, around 7.4) being desired to prevent corneal injury [46]. The EEs of BTB on the different NPs are higher in B-PLGA-95 and B-PCL than in B-PLGA-75, with EE values higher than 60% for B-PLGA-95 and B-PCL and a value of about 27% for B-PLGA-75. A higher encapsulation efficiency with regard to a higher amount of polymer was also observed in the work of Lee and co-workers investigated the effects of polymer concentrations in relation to the drug when preparing NPs, and they observed that, when the polymer to drug ratios increased, maximum EEs were achieved [47]. 

The morphological study realized by TEM shows the distinctive shapes that the three NPs develop once formed, with B-PCL forming more aggregates, which is a drawback as it loses the homogeneity desired in these formulations. Human tears exhibit a non-Newtonian, shear-thinning viscoelastic profile that extends the contact time with the open eye and safeguards the eye’s surface by reducing viscosity throughout the blinking process. Blinking creates significant shear rates within the tear film; as a result, a reduced viscosity is required to prevent harm to the epithelial areas. Viscosity and contact time may be associated with both the comfort experienced and the sharpness of vision. Consequently, these elements could affect patient compliance with their treatment plan and overall quality of life [48,49,50]. With this in mind, we evaluated the viscosity as an essential characteristic of the NPs developed for ocular delivery. Rheology influences the physical stability, ease of product application, spreadability, and residence time of an ophthalmic product. Ideally, an eye drop formulation that is not a solution should possess adequate viscosity to extend contact time on the eye, while also being easily flowable when subjected to shear conditions during blinking, to ensure an even distribution across the surface of the eye [51]. Extensibility is the evaluation of the formulation focused on its ability to spread; in other words, how easily the formulation spreads by blinking. The extensibility studies indicate that the formulations are appropriate for use as eye drops. 

The stability study results showed that the three formulations, which were stored at room temperature and at 4 °C, had no significant differences between the two temperatures after a week. Differences can be observed between the values on the first day and those obtained after a month, and these differences are less significant for the formulations stored at room temperature. Despite the differences observed, these do not cause a negative impact at a critical level since the values are still within the acceptance criteria for stability [43].

Ophthalmic products are required to meet strict sterility standards [52,53]. Ensuring sterility is crucial since microbial contamination can lead to conditions such as conjunctivitis, keratitis, and even infectious endophthalmitis, which may result in vision loss [52]. Our NPs were sterilized via filtration through a syringe filter of 0.22 µm, and a Challenge Test was performed to evaluate the product’s resistance—“robustness”—to microbial contamination during its use. Among the three NPs tested in microbiology studies, B-PLGA-75 NP is the nanoparticle that offers the best results against the harmful effects that contamination or microbial proliferation can cause during storage and use. For the rest, it would be necessary to add a suitable preservative to limit microbial contamination during storage and use or to store them in unidose packaging. In the treatment of pathological conditions, ocular drug delivery systems should ensure that drug molecules reach the site of action at determined concentrations and within the effective therapeutic dosage range. For this reason, a permeation study was performed. The concentration of Transcutol^®^ was optimized to ensure sink conditions while maintaining the integrity of the corneal tissue. This was evaluated upon completion of the permeation study by determining the corneal hydration level. The corneal HL is a parameter frequently used to evaluate the damage to this tissue. Silva-Abreu et al. did an ex vivo cornea permeation with Transcutol^®^/water solution (60/40 *v*/*v*). They assessed the cornea integrity after the experiments by ocular HL, which showed values within the accepted range of 76–80% [28]. HL values 3–7% above the normal value denote damage of the epithelium or endothelium [54]. The HL for the assayed NP formulations was 78.24%, which corroborated the lack of damage on the corneal tissue.

Ex vivo corneas are often used to study drug permeation through the corneal tissue. The goal is to understand how the drug penetrates the cornea and reaches the anterior chamber of the eye after the ophthalmic application. The ex vivo permeation studies show that BTB reached the receptor fluid from all three NPs, suggesting that the NPs could be good carriers for transporting the drug to the aqueous humor. B-PLGA-95 is the formulation that promotes the most diffusion of BTB through the cornea, followed by B-PCL, and finally B-PLGA-75. The permeation enhancement ratio (ER) is a quantitative measure used to compare the permeation of a drug from a nanoparticle formulation to that from a plain solution. An ER greater than 1 indicates that the nanoparticle formulation enhances drug permeation compared to the plain solution. The higher the ER, the more effective the NPs are at enhancing permeation. NPs enhanced the permeation of BTB between 15 and 28 folds. The lag time (*T_l_*) is the time it takes to observe BTB in the steady state, and this is similar for all three NPs. After a *T_l_* of about 3 h, a linear relationship between the cumulative amounts of permeated drug versus time was observed for the assayed NPs, indicating that the permeation rate was constant. Regarding the partition and diffusion coefficients (*P*_1_, *P*_2_, respectively), *P*_1_ describes the ability of the drug to leave the formulation and reach the cornea; the higher the value, the higher this capacity is. The only NP formulation with a significantly higher value is B-PLGA-95. On the other hand, *P*_2_ explains the ability of the drug to diffuse through the cornea; this parameter does not have differences in regard to any of the studied NPs. The amount of BTB retained in the cornea (*Q_ret_*) is statistically different between B-PLGA-95 NPs and B-PCL/B-PLGA-75. The highest drug retention was observed for B-PCL NPs, followed by B-PLGA-95 and, finally, B-PLGA-75. Therefore, the NPs would be in contact with the corneal surface, forming a drug depot from which BTB would be directly transferred.

The histological analysis of the corneas exposed to the NP formulations showed no alteration on the cornea surface. Although in the case of B-PLGA-95, a slight loosening of the substantia propria could be observed, the results of the HET-CAM study indicate that the NPs made from B-PLGA-95, B-PLGA-75, and B-PCL are well tolerated and do not have irritation potential in regard to the eye. This is in line with the biodegradable and biocompatible nature of these polymers, which are known to be well tolerated by ocular tissues [55,56]. The results of the cornea transparency test also support this conclusion, as the corneas treated with B-PCL or B-PLGA-75 had similar transparency to the negative control, further indicating that these NPs are safe for ocular administration. Conversely, corneas treated with B-PLGA-95 showed higher transmittance values compared to the negative control, indicating potential improvement in corneal tissue clarity. 

The results obtained and analyzed in this section help us choose the B-PLGA-95 NPs as the best outlook for future studies. They are ophthalmologically compatible and appear to effectively increase the diffusion of the drug into the cornea, where the drug is retained and could have a local effect.

## 5. Conclusions

The results of this study aim to accomplish the demand for efficient ocular drug delivery systems. This study reports an approach on the use of a three-factor three-level central rotatable composite 2^3+^ star design in the optimization of PLGA and PCL NPs containing BTB using the solvent displacement method. Three different NP formulations were optimized for delivering BTB to the cornea. These NPs were stable for at least 30 days, stored at 25 and 5 °C, and enhanced the permeation of BTB. Among the three NPs, the encapsulation of BTB in PLGA-95 NPs showed the most promising results. In vivo studies should be addressed in the future to demonstrate the advantages of PLGA-based NPs loading BTB for treating ocular diseases.

## Figures and Tables

**Table 1:** Factors and levels in experimental design. Polymer (PCL and PLGA) and surfactant (Poloxamer P188) amounts and aqueous phase volumes.

	Factors					
Variables Levels	Polymer Amount (mg)	Surfactant Amount (mg)	Aqueous Phase Volume (mL)	Polymer Amount (mg)	Surfactant Amount (mg)	Aqueous Phase Volume (mL)
	PCL	PCL	PCL	PLGA	PLGA	PLGA
Low (−1)	50	200	38	75	150	50
Centered (0)	60	240	44	95	200	60
High (+1)	70	280	50	115	250	70
Axial (−0.39)	46	185	36.5	72	134	47
Axial (+1.39)	74	295	51.5	118	266	73

**Table 2:** Resulting formulations of the 2^3+^ star central composite rotatable factorial design based on three levels (low (0), central (0.5) and high (1)) for each factor: amount of Polymer (PLGA or PCL), amount of Surfactant (P-188), and the volume of the aqueous phase.

Levels of Factors			
Formulations	Polymer Amount (mg)	Surfactant Amount (mg)	Aqueous Phase Volume (mL)
F1	0	0	0
F2	1	0	0
F3	0	1	0
F4	1	1	0
F5	0	0	1
F6	1	0	1
F7	0	1	1
F8	1	1	1
F9	0.5	0.5	0.5
F10	0.5	0.5	0.5
F11	−0.39	0.5	0.5
F12	1.39	0.5	0.5
F13	0.5	−0.39	0.5
F14	0.5	1.39	0.5
F15	0.5	0.5	−0.39
F16	0.5	0.5	1.39
F17	0.5	0.5	0.5
F18	0.5	0.5	0.5
F19	0.5	0.5	0.5

**Table 3:** Composition of the selected formulations, each containing 10 mg of BTB.

Formulation	Polymer Amount (%)	Aqueous Phase (%)	Surfactant Amount P188 (%)
B-PLGA-95	0.157	99.511	0.332
B-PLGA-75	0.107	99.609	0.284
B-PCL	0.100	99.502	0.398

B-PLGA-95: prepared with 95 mg of poly(lactic-co-glycolic) acid; B-PLGA-75: prepared with 75 mg of poly(lactic-co-glycolic) acid; B-PCL: prepared with 50 mg of poly(ε-caprolactone); P188: Poloxamer 188.

**Table 4:** Microorganisms used to conduct the microbiological tests.

Test Microorganisms			Microorganism Concentration	
			B-PCL NPs (CFU/mL)	B-PLGA-95 and B-PLGA-75 NPs (CFU/mL)
Bacteria	*Pseudomonas aeruginosa*	ATCC 9027 (NCIMB 8626, CIP 82.118)	1.5 × 10^8^	2.3 × 10^8^
	*Staphylococcus aureus*	ATCC 6538, (NCTC 10788, NCIMB 9518)	2.1 × 10^8^	2.3 × 10^8^
Fungus	*Candida albicans*	ATCC 10231 (NCPF 3179, IP 48.72)	1 × 10^8^	1 × 10^8^
	*Aspergillus brasiliensis*	ATCC 16404 (IMI 149007, IP 1431.83)	2 × 10^7^	2 × 10^7^

Tests were conducted with each microorganism individually. CFU: colony forming units.

**Table 5:** Criteria for ophthalmic formulations.

Log Reduction (CFU/mL)						
	Criteria	6 h	24 h	7 d	14 d	28 d
Bacteria	A	2	3	-	-	NR
	B	-	1	3	-	NI
Fungus	A	-	-	2	-	NI
	B	-	-	-	1	NI

NR: Not found; NI: Not increased.

**Table 6:** Criteria for classification of the severity of irritation [30].

Irritation Score	Irritation Classification
0–0.9	Non-Irritant
1–4.9	Slight Irritant
5–8.9	Moderate Irritant
9–21	Severe Irritant

**Table 7:** Responses for each PLGA-based formulation: mean particle size (Z-ave) and polydispersity index (PDI).

Levels of Factors				Responses	
Formulations	PLGA Amount (mg)	P188Amount (mg)	Aqueous Phase Volume (mL)	Z-Ave ± SD (nm)	PDI ± SD
F1	75	150	50	206.65 ± 2.9	0.303 ± 0.016
F2	115	150	50	245.80 ± 2.1	0.177 ± 0.035
F3	75	250	50	201.50 ± 1.8	0.265 ± 0.049
F4	115	250	50	237.55 ± 5.0	0.150 ± 0.001
F5	75	150	70	180.80 ± 4.9	0.213 ± 0.050
F6	115	150	70	209.90 ± 3.0	0.231 ± 0.035
F7	75	250	70	197.25 ± 1.2	0.217 ± 0.053
F8	115	250	70	233.05 ± 1.3	0.166 ± 0.079
F9	95	200	60	98.20 ± 0.0	0.090 ± 0.056
F10	95	200	60	118.70 ± 0.6	0.168 ± 0.017
F11	72	200	60	209.55 ± 1.8	0.103 ± 0.028
F12	118	200	60	288.20 ± 4.5	0.091 ± 0.051
F13	95	134	60	214.75 ± 3.6	0.218 ± 0.022
F14	95	266	60	225.25 ± 3.0	0.117 ± 0.009
F15	95	200	47	217.55 ± 2.2	0.251 ± 0.008
F16	95	200	73	206.40 ± 1.6	0.101 ± 0.038
F17	95	200	60	116.60 ± 0.7	0.184 ± 0.002
F18	95	200	60	104.90 ± 0.0	0.191 ± 0.016
F19	95	200	60	159.75 ± 0.8	0.056 ± 0.010

The highlighted formulation was one of the selected ones for further studies.

**Table 8:** Responses for each PCL-based formulation: mean particle size (Z-ave) and polydispersity index (PDI).

Levels of Factors				Responses	
Formulations	PCL Amount (mg)	P188 Amount (mg)	Aqueous Phase Volume (mL)	Z-Ave ± SD (nm)	PDI ± SD
F1	50	200	38	104.20 ± 1.70	0.137 ± 0.004
F2	70	200	38	138.30 ± 5.94	0.165 ± 0.016
F3	50	280	38	109.35 ± 0.78	0.174 ± 0.006
F4	70	280	38	120.00 ± 6.93	0.247 ± 0.062
F5	50	200	50	127.50 ± 1.98	0.107 ± 0.037
F6	70	200	50	398.65 ± 15.34	0.159 ± 0.047
F7	50	280	50	366.40 ± 4.38	0.160 ± 0.065
F8	70	280	50	339.75 ± 2.76	0.197 ± 0.009
F9	60	240	44	347.50 ± 2.55	0.223 ± 0.015
F10	60	240	44	426.60 ± 6.79	0.029 ± 0.030
F11	46	240	44	403.90 ± 1.56	0.096 ± 0.053
F12	118	240	44	335.75 ± 3.32	0.355 ± 0.066
F13	60	185	44	370.60 ± 4.53	0.158 ± 0.031
F14	60	295	44	349.95 ± 6.43	0.182 ± 0.040
F15	60	240	36.5	401.35 ± 7.85	0.215 ± 0.011
F16	60	240	51.5	374.95 ± 1.06	0.200 ± 0.013
F17	60	240	44	458.10 ± 4.95	0.203 ± 0.027
F18	60	240	44	356.90 ± 2.83	0.181 ± 0.034
F19	60	240	44	309.70 ± 6.08	0.147 ± 0.048

The highlighted formulation was one of the selected ones for further studies.

**Table 9:** Physicochemical properties of the optimized NPs: B-PLGA-95, B-PLGA-75, and B-PCL NPs. All measurements have been taken with three replicates (*n* = 3).

Formulation	Z-Ave ± SD	PDI ± SD	ZP (mV) ± SD	pH ± SD	EE (%) ± SD
(NPs)	(nm)				
B-PLGA-95	84.50 ± 0.72	0.10 ± 0.01	−25.70 ± 2.01	6.9 ± 0.3	65.63 ± 3.87
B-PLGA-75	77.65 ± 0.03	0.04 ± 0.02	−28.20 ± 0.14	6.8 ± 0.3	27.39 ± 2.05
B-PCL	138.36 ± 0.92	0.09 ± 0.02	−20.55 ± 0.07	6.8 ± 0.2	77.82 ± 5.33

Z-ave: average particle size; PDI: polydispersity index; ZP: zeta potential; SD: standard deviation; EE: Encapsulation efficiency.

**Table 10:** Mathematical fitting of the NPs extensibility, which followed the two-site binding parabola model. Results are reported as the mean of each model parameter and the standard error (Std. Error).

Best-Fit Values	B-PCL	B-PLGA-75	B-PLGA-95	Water
A	16.03	−5.633	2.408	31.99
B	0.125	0.562	0.624	0.812
C	−0.000279	−0.002398	−0.003030	−0.003556
Std. Error				
A	0.456	2.849	3.556	10.720
B	0.016	0.097	0.121	0.365
C	0.000101	0.000629	0.000785	0.002366
Goodness of Fit				
R^2^	0.999	0.994	0.988	0.960

**Table 11:** Physical stability study of NP formulations after 30 d (*n* = 3) of storage at 25 and 5 °C. (Mean ± SD).

NPs	Storage Condition (°C)	Z-Ave (nm)			PDI		
		Time Assayed (Days)					
		0	7	30	0	7	30
B-PLGA-95	25	80.56 ± 0.72	81.72 ± 0.22	83.03 ± 2.90	0.03 ± 0.01	0.07 ± 0.02 *	0.07 ± 0.05 *
	4	82.24 ± 0.54	85.48 ± 0.72	91.07 ± 4.85 *	0.02 ± 0.01	0.03 ± 0.01	0.07 ± 0.08 *
B-PLGA-75	25	71.43 ± 0.01	74.75 ± 2.24	81.42 ± 5.42 *	0.05 ± 0.01	0.07 ± 0.02	0.08 ± 0.04
	4	75.23 ± 0.15	77.58 ± 0.24	86.00 ± 5.24 *	0.02 ± 0.01	0.04 ± 0.02	0.06 ± 0.01 *
B-PCL	25	130.36 ± 1.82	134.8 ± 2.12	141.5 ± 5.03 *	0.03 ± 0.01	0.07 ± 0.04	0.12 ± 0.08 *
	4	128.30 ± 1.23	131.30 ± 2.82	152.5 ± 7.79 *	0.06 ± 0.02	0.09 ± 0.07	0.02 ± 0.04 *

* Statistical differences versus Time Assayed = 0 d (*p* < 0.01).

**Table 12:** Criteria based on Log reduction for microorganisms by B-PLGA-95, B-PLGA-75, and B-PCL NPs.

Microorganism	Nanoparticle	Log Reduction (CFU/mL)				
		6 h	24 h	7 d	14 d	28 d
*P. aeruginosa*	B-PLGA-95	1	3	NR		NR
	B-PLGA-75	NR	NR	-	-	NR
	B-PCL	1	NI **	3	-	NR
*S. aureus*	B-PLGA-95	1	NR *	NR		NR
	B-PLGA-75	NR *	NR	-	-	NR
	B-PCL	-	1	NI (10^5^)	-	NR
*C. albicans*	B-PLGA-95	-	-	NI	1	NI
	B-PLGA-75	-	-	3	NI **	NI
	B-PCL	-	-	NI (10^6^)	~1	NI
*A. brasiliensis*	B-PLGA-95	-	-	1	NI (1)	NI
	B-PLGA-75			NR	NR	NR
	B-PCL	-	-	NI (10^5^)	NI (10^5^)	NI

* NR: not found; ** NI = no increase.

**Table 13:** Permeation parameters from B-PLGA-95 NPs, B-PLGA-75 NPs, and B-PCL NPs expressed by the mean and standard deviation of six replicates (*n* = 6). Predicted plasma concentration at the steady-state (*C_ss_*); SD = standard deviation; b.l.q. = below the limit of quantification. The statistical analysis consisted of one-way ANOVA test followed by Tukey’s Multiple Comparison Test (*p* < 0.05) expressed with letters: ^A^ = statistically significant differences between with B-PLGA-95; ^B^ = statistically significant differences with B-PLGA-75; ^C^ = statistically significant differences with B-PCL.

		B-PLGA-95	B-PLGA-75	B-PCL	B-Solution
Parameters	Units	Mean ± SD	Mean ± SD	Mean ± SD	Mean ± SD
Flux (*J*/cm^2^)	µg/h/cm^2^	20.90 ± 2.47	10.12 ± 1.23 ^A^	16.12 ± 1.92 ^A,B^	0.07 ± 0.01 ^A,B,C^
Permeability coefficient (*K_p_*)	cm/h	0.126 ± 0.014	0.071 ± 0.008 ^A^	0.081 ± 0.009 ^A^	0.004 ± 0.000 ^A,B,C^
Lag Time (*T_l_*)	h	2.90 ± 0.53	2.75 ± 0.37	2.84 ± 0.24	0.83 ± 0.07 ^A,B,C^
Partition coefficient (*P*_1_)	cm	2.19 ± 0.02	1.17 ± 0.02 ^A^	1.38 ± 0.01 ^A,B^	0.03 ± 0.00 ^A,B,C^
Diffusion coefficient (*P*_2_)	1/h	0.057 ± 0.007	0.061 ± 0.005	0.059 ± 0.005	0.139 ± 0.014 ^A,B,C^
Amount of BTB in the skin (*Q_ret_*)	µg/cm^2^	3.35 ± 0.29	0.40 ± 0.04 ^A^	6.09 ± 0.57 ^A,B^	b.l.q.
Enhancement ratio (*ER*)	-	27.7	15.6	17.8	-

## Data Availability

The data are not publicly available due to this work is part of a doctoral thesis, and the data included in this work will be available once the project is completed and presented.
